# Aorto‐cutaneous fistula from an infected ascending aorta graft resulting in massive hemorrhage after a Valsalva maneuver for a heavy weight lift

**DOI:** 10.1002/ccr3.3089

**Published:** 2020-07-09

**Authors:** Alexandros Triantafyllidis, Aikaterini Paraskeva, Konstantinos A. Boulas, Maria Nathanailidou, Konstantinos Chatzipourganis, Anestis Hatzigeorgiadis

**Affiliations:** ^1^ Department of General Surgery General Hospital of Drama Drama Greece

**Keywords:** aortic dissection, aorto‐cutaneous fistula, deep sternal wound infection, hemorrhage, prosthetic replacement, Valsalva maneuver

## Abstract

In the setting of an infected prosthetic ascending thoracic aorta, prompt and definitive surgical treatment is mandatory to avoid catastrophic bleeding complications.

## Graphical abstract

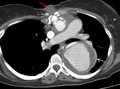

A 69‐year‐old female transported to the emergency department with class IV hemorrhagic shock. Ambulance paramedics found her lay down lethargic covered by severe amount of blood. The patient underwent ascending aorta replacement 2 years prior to a Stanford A acute aortic dissection complicated by deep sternal wound infection including a chronic draining sinus tract and an aorto‐cutaneous fistula at the upper body sternum developed 1 year and 3 months prior, respectively. During resuscitation, external opening compression sutures of the aorto‐cutaneous fistula placed, and the patient stabilized and underwent whole‐body CT with no evidence of free aortic rupture, as shown in Figure 1.

**FIGURE 1:**
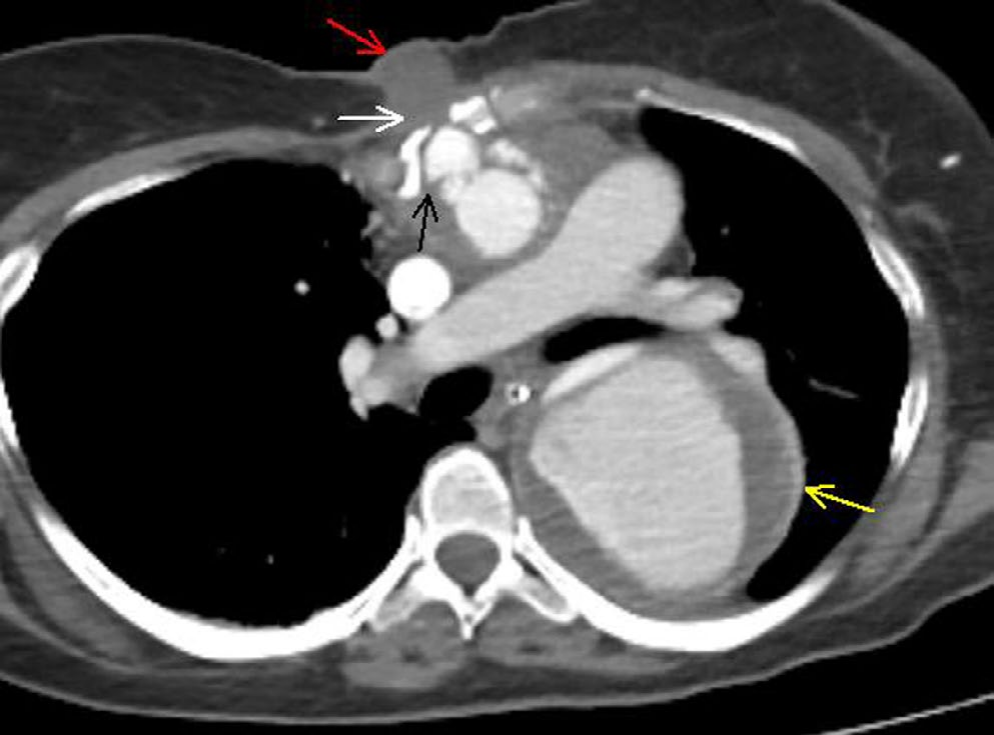
Chest CT angiography showing the aorto‐cutaneous fistula (red arrow). Note the sternal dehiscence (white arrow), the pseudoaneurysm, and surrounding hematoma between the prosthetic ascending aorta and the back of the sternum (black arrow) along with the aneurysmatical dilatation of the false lumen of the thoracic aorta after the prosthetic replacement (yellow arrow), which were unaltered compared to follow‐up CTs. There was no evidence of free thoracic aorta rupture

## 1 QUIZ QUESTION: WHAT WENT WRONG?

According to family, the patient performed a heavy weight lift before exhibiting oozing blood from the aorto‐cutaneous fistula. As there was no adequate patient and family education, local bleeding management by firm pressure to the external opening was not performed resulting in severe hemorrhage. The Valsalva maneuver that the patient performed for weight lift resulted in transient aortic pressure increase which normally has no significant impact. However, in the setting of an infected prosthesis combined with false aneurysm and aorto‐cutaneous fistula, transient significant aortic pressure increase led to aortic rupture.^1^ In analogous cases, patient education for avoiding Valsalva maneuvers and local bleeding control until definitive surgery is crucial. However, redo sternotomy is complex and associated with considerable morbidity and mortality. General principles are as follows: (a) safe re‐entry by peripheral cannulation with or without cardiopulmonary bypass; (b) removal of the infected graft, debridement of the infected periprosthetic tissues, vascular reconstruction, and long‐term antibiotic therapy.^2^

## CONFLICT OF INTEREST

The authors declare that they have no conflict of interests.

## AUTHOR CONTRIBUTIONS

All authors equally accessed the data and contributed to the preparation of the manuscript. TA and HA: responsible for making and performing treatment decisions. HA: reviewed the manuscript for critical intellectual content and had the final approval.

## INFORMED CONSENT

Informed consent was obtained from the patient.

## STATEMENT OF HUMAN AND ANIMAL RIGHTS

The present article does not contain any studies with human or animal subjects performed by any of the authors.
